# The First Signs of Insanity, Their Prevention and Treatment

**Published:** 1913-03

**Authors:** 


**The First Signs of Insanity, their Prevention and Treatment.** By BERNARD HOLLANDER, M.D. Pp. 347. London: Stanley Paul & Co. [1912.] 10s. 6d. net.

There is no inconsiderable output of books written by medical men on medical subjects, addressed to the laity and published through the lay press. The explanation is perhaps to be found in an altruistic spirit which yearns to educate this class. The instruction may be in the matter of their feeding and digestion, in the understanding and management of their "nerves;" in some the mysteries of sexual life are conveyed with a pornographic sauce suitable to salacious palates; whilst most convey a very evident revelation of the author's powers of insight, judgment and treatment. Nor is the address of his consulting-rooms unknown as an affix to the preface.

In the matter of anything that might appeal to the morbid or superficial reader Dr. Hollander's book is quite free. Its purpose as set forth is to show what insanity really is, how it can be prevented, and how treated before it reaches the incurable stage; no small task, we should opine. It may at once be said that the work is a good and useful compilation of all that is necessary to place before the laity, and is expressed in suitable language. We assume the intention of the work to be mainly popular (although the general practitioner and lunacy specialist are also instructed), not only because so stated by the author, but because there is a certain suggestion not wholly absent as to Dr. Hollander's acumen and success in diagnosis, prophylaxis and treatment such as would be needless in a work addressed merely to his own profession. In a book so destined a certain freedom of detail and expression is allowable, but there are to be found many statements which cannot be allowed to pass without comment. We might be led to infer, for instance, from the allusion to the "non-recognition of the principles of localisation of mental functions," that no investigations had been made, no speculations advanced, no conclusions entertained in this matter; whereas it is only the almost insuperable difficulties in arriving at any sound inferences that has prohibited the real scientific worker from so doing. One of the foremost of these has written to the effect that practical psychiatry has not yet begun to consider the distribution in the cortex of the purely psychical functions as distinct from sensory and motor processes. Dr. Hollander reminds us that "behind those sensory and motor centres there lie also all the functions which constitute mental phenomena." Very much behind, we agree, if not taken in a topographical sense. If Dr. Hollander can indicate any reliable and corroborated proof of this localisation other than has been furnished by Wundt, Munk, Monakow, Tanzi and others they will be welcome. We are not unmindful of his work on this subject so frequently alluded to in the present volume. Certainly in the passages following his quoted remarks we find nothing illuminating. We are in another place advised that more attention should be paid to the shape of the head, both in the living and the dead, and that in the author's opinion the study of abnormal heads in the insane will prove valuable. Such observation has been going on for many decades, and the results have been so indeterminate that no conclusions of value have been drawn. It is true that some observers have declared that though irregularities in cranial conformation are not uncommon in the sane, they are relatively more frequent in the insane ; this is, however, not a matter for the throwing out of conjectures, but for series of measurements and analysis of cases. At any rate, Tanzi does not express himself as sanguine of any results in this direction. He writes, " The cranium is still the subject of measurements and studies as minute as they are sterile in practical results."

Again, Dr. Hollander comments on the profound ignorance existing as to the naked-eye appearance of the brain and the relative value of development of its different parts. Does he not acknowledge the valuable work of Mickle, of Poggi, Giacomini, Mingazzini, Benedikt, not to speak of Bolton, Lattes and others ? At the Wakefield Asylum, and no doubt at others, paraffin casts were preserved of most brains for many years and convolutionary anomalies noted in the records ; so, at least, investigation has not been idle. Hollander has much to say on the subject of surgical interference in cases of cranial traumatic insanity when so determined, and quotes his previous writings in advocacy of the need for this to be adopted with greater frequency than now thought requisite. We think those cases of genuine insanity which are said to have benefited by operation require very careful scrutiny. There certainly are instances, generally admitted, where definite symptoms of brain disturbance, not necessarily insanity, but attended with emotional disturbance or blunting, result from direct or secondary irritation. Excluding gross lesion by laceration, hemorrhage, atrophy, etc., relief afforded by operation is to be hoped for. But the larger number of so-called traumatic insanities are only psychoses in predisposed persons aroused by or associated with accident. Sometimes both psychosis and its improvement by operation are the result of suggestion. That there is no widespread resort to operative interference except in few and carefully chosen cases is not, as might be gathered, owing to supineness or ignorance.

We have no desire to offer too carping a criticism of this book, which contains a great deal that is interesting and profitable to its lay readers ; but we deprecate the tendency shown in many places to set forth, as from a point of vantage, what ought to be done and what has not been done in matters of morbid psychiatry by others than the author, as well as an insufficient recognition of that already achieved. Readers might infer that there was no host of able investigators, the restriction of whose output was that attending all practical and lasting scientific work, as distinguished from the mere compilation of books and general dissertations which neither merit nor require notice.

The book is well produced. We note that almost every public journal seems to have received a copy for review, and that Dr. Hollander's name is duly honoured throughout the land.

